# Filtering Efficiency of Sustainable Textile Materials Applied in Personal Protective Face Mask Production during Pandemic

**DOI:** 10.3390/ma16030903

**Published:** 2023-01-17

**Authors:** Attila Géczy, Gergő Havellant, Réka Bátorfi, Agata Skwarek, Karel Dušek, David Bušek, Balázs Illés

**Affiliations:** 1 Department of Electronics Technology, Faculty of Electrical Engineering and Informatics, Budapest University of Technology and Economics, H-1111 Budapest, Hungary; 2 Department of Electrotechnology, Faculty of Electrical Engineering (K13113), Czech Technical University in Prague, Technická 2, 1902/2, Praha 6, Dejvice, 166 27 Prague, Czech Republic; 3 Łukasiewicz Research Network-Institute of Microelectronics and Photonics, 30-701 Kraków, Poland

**Keywords:** filtering efficiency, personal protective equipment, textile mask, respirator, laser-based particle counting, textile material, COVID-19

## Abstract

The COVID-19 outbreak increased demand for personal protective respirator masks. Textile masks based on cloth materials appeared to be a sustainable, comfortable, and cost-effective alternative available in global communities. In this study, we used laser-based particle counting for mask material qualification to determine the concentration filtering efficiency in general, everyday community use. The efficiencies of eleven different commercially available textile materials were measured in single-, double-, and triple-layer configurations according to their grammage, mesh (XY), and inter-yarn gap. It was found that in the single-layer configurations, most materials were well below the acceptable standards, with a wide variation in filtering efficiency, which ranged from 5% to ~50%. However, when testing the fabrics in two or three layers, the efficiency increased significantly, exceeding or approaching the standard for medical masks. Three layers of natural silk was able to produce a level of filtration efficiency of 84.68%. Two-layered natural silk achieved 70.98%, cotton twill achieved 75.6%, and satin-weave viscose achieved 69.77%. Further options can also be considered in cases where lower filtration is acceptable It was statistically shown that applying a second layer was more significant in terms of overall filtering than increasing the layer count to three. However, layer stacking limited the breathability. The paper presents measurement-based qualitative and quantitative recommendations for future textile applications in face mask manufacturing.

## 1. Introduction

Masks and other various protective measures, such as keeping a distance and continuous hand disinfection, play an important role in limiting the spread of a pandemic (such as SARS-CoV-2). The use of different masks is common practice, but there is also resistance in the community, as the effectiveness and non-convenience of masks are difficult to communicate scientifically. However, masks worn by the masses have been a useful and low-cost addition to social distancing during the COVID-19 pandemic. It shifted the focus from self-defense to the community-focused behavior of citizens [1]. While different masks are available commercially, professional types are mainly produced using different plastic-based layers. Communities have also used cheap, reusable, and sustainable cloth-based textile masks to improve personal protection against poor air quality and to reduce viral spread—this study aimed to investigate these masks, as sustainable consumption is key for future global communities. Qualitative and quantitative analyses are limited for a wide range of materials, so the materials must be investigated more thoroughly.

The viral protection capabilities of masks have been studied for years, but the results have constantly been reinterpreted with the onset of the COVID-19 pandemic. In a 2006 study, the results showed [2] that masks with an exemplary N95 certification do not necessarily provide complete protection against viruses under the accepted minimum penetration-prone particle size of 300 nm. Thus, the protection against various airborne viral pathogens provided by some N95 masks may fall below 95%, especially at higher inhalation flow rates. The situation is different for SARS-CoV-2 strains—while these viruses are primarily spread by large (>1–10 μm) respiratory droplets, aerosols can be carried horizontally up to 2 m height and then fall to the ground within seconds. Even surgical masks may be able to provide adequate protection in this droplet size range [3].

It is recognized that the use of masks is helpful in slowing down an epidemic. Li et al. considered three key factors [4] that contribute to the effectiveness of mask wearing in reducing transmission risk, including the aerosol reduction rate of the mask, the adoption of masks in the population, and the general availability of masks. Li reinforced the previous idea [4] that the face mask can be effectively combined with social distancing to flatten the epidemic curve. Textile masks, i.e., cloth masks, have also been studied recently, but with general qualitative analysis-based discussions, missing deeper statistical analysis [5]. It was noted that until the cloth mask design is proven to be as effective as a medical or N95 mask, the wearing of fabric masks cannot be recommended for healthcare workers. However, the article notes that in a residential setting, textile masks can actually be used to prevent or control the spread of infections. According to [5], the protection provided by textile masks can be improved by selecting the right material, increasing the number of mask layers, and using masks with a design that provides the better filtration and fit. Textile masks can be washed daily and after high exposure with soap and water or other appropriate methods; however, an in-depth discussion was not detailed in the paper. Commercially available (store-bought) masks were evaluated by Maurer et al. [6] for filtration and air resistance, and reusability was also considered. It was found that in the case of commercial masks, high filtration efficiency is associated with high air resistance and vice versa. The filtration efficiency should be chosen as high as possible, considering the wearer’s tolerance to air resistance. Ho et al. [7] suggested that textile masks could be potential substitutes for a surgical mask for a person with a respiratory tract infection in an air-conditioned microenvironment. Healthy people can use textile masks daily in the community, also they are washable and reusable. In their work, they also considered the application of three layers [7]. Textile-based community masks were declared to have low to no filtration efficiency by Sousa-Pinto et al. [8]; these materials were perceived to be more of a hygienic measure that minimizes the projection of the user’s respiratory droplets, saliva, sputum, and respiratory secretions when talking, coughing, or sneezing. The filtration efficiency of woven fabric was also investigated using a 3D modeling approach. Rios de Anda et al. found [9] that the presence of inter-yarn gaps causes weak filtration below and above the micrometer range. They reported a filtration performance as weak as 2.5–10% filtration. Their conclusion summarizes that it may be impossible to prepare acceptable filters from single-woven fabrics. According to the recent literature, the results are not straightforward, and most papers have missed the opportunity for a detailed, quantitative, and statistically established analysis of these materials.

Flame photometry can be used for mask validation [10,11], but the apparatus is complex, and the method is difficult to use efficiently. A laser-based particle counting method was recently developed [12] to measure masks in an efficient workflow. Other particle counting methods have also been applied for the cause. Rengasamy et al. [13] successfully compared N95 respirators using photometric and ultrafine condensation particle counting (UCPC) methods. Kim et al. [14] successfully applied the UCPC method to detect the penetration of nanoparticles (3–20 nm) on commercial filter media. However, the UCPC method is limited in terms of the flow rate [15]. Other complex solutions can be found in the literature, but due to their extended apparatus requirements, they are far from being effective.

Based on the previous findings and the lack of deep quantitative and qualitative analyses on mask material selection, this paper presents a study on the particle filtration efficiency of commercially available, sustainable textile materials [16] with a fast, low-cost, and efficient mask validation method [12]. The qualitative analysis reveals more information about inexpensive community masks made of textile materials and highlights the limitations of different types, which were commercialized during the years of the pandemic. While the most recent study highlighted very weak filtration capabilities of limited types of cloth-based textile materials [17], the World Health Organization (WHO) suggests that wearing a poor filtering cloth is still a better alternative than not wearing anything [18]. The problem needs a thorough analysis of a varied selection of materials and a statistical analysis, which was also performed in this study. It must be noted that while comparative assumptions are discussed in the paper with the EN 149:2001 in mind, our results are not in full accordance with the given depth and comprehensive approach of the standard. We mostly focus on a fast and efficient measurement methodology that can assess community needs regarding the choice of material for personal protection.

## 2. Materials and Methods

### 2.1. Classification of Protective Masks and Respirators

For the qualification of masks or respirators (such as personal protective equipment—PPE [19]), different systems of standards have been developed depending on the specifics of the area/country. The three major systems are filtering facepiece particle (FFP), non-oil (N), and the Chinese “Kǒuzhào” non-oil standard (KN). The FFP 1, 2, and 3 masks of the European Union standard usually have the following filtering efficiencies: 80%, 94%, and 99%. The American versions [19,20] are the N95 and N100, which have 95% and 99.97% filtering efficiencies. According to the Chinese classification, KN95 masks can achieve 95% filtration. The differences among countries also lead to some variation in the definition of the filtering efficiency, but an acceptable approximation is that the FFP2, N95, and KN95 [21] can be considered the same. Medical (or surgical) masks typically have an efficiency of 67%.

### 2.2. Textile Material Types

Textile cloths are reasonable sources to fabricate handmade masks in small-scale production. In the population, many people turned to these protection methods. We obtained materials that have actually been applied to make commercial masks in the past year for people. The 11 fabrics shown in Table 1 were used in the tests.

The fabrics were classified according to weight, mesh density (the number of fibers within an area of 1 inch), and inter-yarn gap size. The materials were investigated in single-, double-, and triple-layer stacked configurations.

### 2.3. Textile Material Classification Methods

The fabric weights of the samples were measured using an A&D HM-300 scale. The grammage, or fabric weight (grams per square meter—GSM, g/m^2^), was chosen as the most common and comparable value of the textiles. The fabric weight is an essential parameter of fabrics and gives a numerical value that characterizes the thickness and density among weave types (plain, twill, satin, etc.) or knitting patterns (courses and wales, weft and warp, etc.). A higher grammage value can be reached by two means: a denser pattern made of thinner yarns or a loose pattern with thicker yarns.

For optical analysis, an Olympus SZX9 (Japan) microscope with a digital camera was used for the thread inspection. The diameter of the fiber was calculated from the microscope image. The inter-yarn pore size (inter-yarn surface) was also determined. The material pieces were chosen randomly from A4-sized sheets. The XY mesh was calculated according to the photos, where the usual yarn count was in the range of ~10—the mesh size was calculated according to the scale bar and the pixel count. The inter-yarn surfaces (as showed later in Figure 5) were also calculated using the same image processing based on 15-15 located pores by defining the area not covered by fibers in the textile and measuring the average height and weight of the apertures. If the pore size is large, the filtration efficiency is likely to be reduced [9].

### 2.4. Filtration Efficiency Measurement

**Figure 1:**
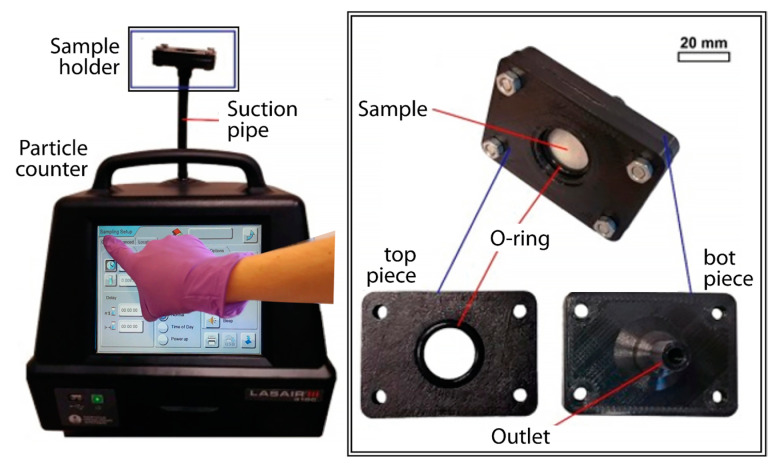
The setup of our particle-counting-based measurement system.

The filtration efficiency of the different textiles was determined by a method developed by the authors, which is based on laser particle counting [12]. The main parts of the measurement system are a particle counter (LASAIR III 310C, coincidence error below 10%) and a sample holder that holds the textile in place during the measurements. The sample holder can hold a sample with a 30–35 mm diameter. An O-ring blocks leakage during the measurement. The sample holder is connected to the particle counter with a suction pipe. For the measurements, the samples were placed on the O-ring between the bottom and upper fixtures. The setup is shown in Figure 1.

The Lasair III 310C is a portable aerosol particle counter that is usually used to qualify clean-room laboratories in microelectronics or medicine production. It works with a laser-based counting method. The accessing particle in the device passes through laser light. A photodetector detects the redirected light and the loss of light, which determines the size of the obstructing particle. The particle counter distinguishes the counted particles according to the estimated sizes into 6 channels: 0.3–0.5 µm, 0.5–1 µm, 1–5 µm, 5–10 µm, 10–25 µm, and >25 µm. The volume flow rate of the particle counter was 30 L/min, which is approximately the average breathing flow of humans in comfortable conditions.

The measurement involved two steps. First, the particle number concentration (PNC) (piece/m^3^) of the ambient air was measured for 1 min. Right after, the sample holder was connected to the particle counter, and the PNC behind the filter material was measured again for 1 min. The filtering efficiency was determined using the PNC differences [11]. With our method, the concentration filtering efficiency (*CFE*, %) (1) can be measured and calculated for any particle size range of the measurements:

(1) $$CFE=\left(\frac{PC_{A}-PC_{C}}{PC_{A}}\right)\times 100,[\%]$$

where *PC_A_* is the particle concentration of the ambient air (mg/m^3^), and *PC_C_* is the particle concentration behind the textile (mg/m^3^). The particle counter can measure particle number concentrations (PNCs) directly; the particle concentrations (PCs) can be calculated from the PNCs and the particle sizes. It was assumed that the specific density of the particles in the ambient air was nearly homogeneous. The EN 149:2001 standard defines *CFE* values in the case of aerosols to have 0.4 and 0.6 µm median mass aerodynamic diameters (MMAD). Our previous analysis of the MMAD parameter of ambient air showed that in the particle range between 0.3 and 5 µm, the MMAD was between 0.34 and 0.76 µm. This is close to the requirements of the EN 149:2001 standard. Therefore, the particle concentrations (PC) were counted only from the 0.3–5 µm range during the CFE calculations [12]. The machine is not capable of 0.1 µm measurements to serve as a baseline for higher FFP comparison; however, this is only necessary at the N95 level [22], and as the results show, the textiles were below this level. Additionally, the problem with SARS-CoV2 is particle transport is above 1 µm range [3,22]. It needs to be clarified that the use of atmospheric aerosols is not in line with the EN 149:2001 standards, but they can supply practical results, which are in line with the results obtained from standard-validation-based mask materials [12]. Further discussion on the repeatability, efficiency, variation in efficiency, and further measurement parameters can be found in [12].

The upper edge of the defined breathability was given as 30 L/min [12]. It has to be noted that the airflow resistance was not measured directly. The 30 L/min value is close to the comfortable limit, as it is higher than usual breathing, and it is suggested that in case of lower volume flow (normal breathing), the efficiency will not be worse, as we reported before in [12]. To sum up, our case focuses on at-rest (6 L/min), normal activity (16 L/min), and light exercise scenarios (where the minute ventilation of moderate exercise can be defined as 40 L/min) [23]. It can be said that for exercise scenarios or more intense activities, other approaches are needed. However, textile masks are mostly used in general community activities, not for sports or moderate to extreme physical work.

For the statistical analysis to show the statistically significant differences among the samples, single-factor variance analysis and Tukey’s test were performed using Statistica software (TIBCO Software Inc., Palo Alto, CA, USA) at a significance level (α) of 0.05.

The Pearson coefficients were calculated in Excel to show the correlations between the filtration efficiency and the grammage, mesh (XY), and inter-yarn pores.

## 3. Results

### 3.1. Obtained Textile Material Parameters

The optical measurements were based on the photos shown in Table 2. The plotted values presented later are averages based on 5-5 measurements.

**Figure 2:**
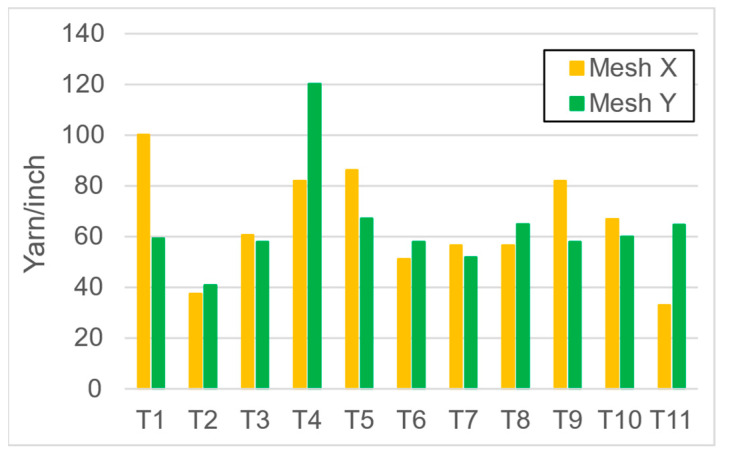
Measured mesh numbers on T1-T11 samples.

The mesh numbers were roughly the same in the XY direction, with a few exceptions (Figure 2, e.g., twill, satin viscose, linen canvas). The inter-yarn pores (presented later in Figure 5) were minimal for some textiles (T1, T4, T5, T9, and T11) and significant for others (T2, T3, T6, T7, T8, and T10). The following results were recorded for the latter group, showing significant standard deviance (variation in pore size): T2 (0.018 mm^2^, σxy = 0.019); T3 (0.035 mm^2^, σ = 0.017); T6 (0.009 mm^2^, σ = 0.009); T7 (0.033 mm^2^, σ = 0.017); T8 (0.011 mm^2^, σ = 0.013); T10 (0.016 mm^2^, σ = 0.010). Presumably, fabrics without inter-yarn pores will function as good filtering materials. T1 already had a considerable pore dimension in the ~10 micrometer range. This dimension is comparable to aerosol particles with 1–10 μm size [3,21], which is the size range capable of transmitting COVID-19. At this point, it can be assumed that cotton canvas or knitted fabrics will perform poorly, especially those with large inter-yarn pores (e.g., linen fabrics, mercerized cotton canvas). Colors in Table 2 represent the original colors of the materials.

### 3.2. Filtration Efficiency Results

**Figure 3:**
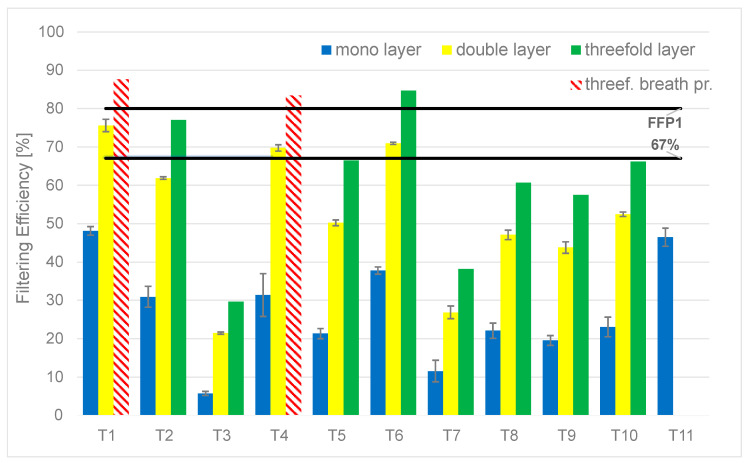
Filtration efficiency of single-, double-, and triple-layer filtration efficiency, where the 67% lower boundary of surgical mask and the higher boundary of FFP1 masks is highlighted. The cross-hatched bars represent the measurements at the upper edge of defined breathability (30 L/min) in triple-layer configurations.

Figure 3 shows the results of filtration efficiencies of the single-, double- and triple-layer fabrics. The thresholds of surgical masks and FFP1 masks are indicated by the horizontal lines. The original single-layer configuration was usually weak in filtration, with the quantitative results ranging from 5.7–48.1% in the investigated textiles. T1 and T11 were outstanding in this aspect, followed by T4 and T6. The filtration efficiency improvement between one and two layers was twofold on average. The average improvement after adding a third layer was 27% on average. However, the three layers might be ergonomically problematic due to breath comfort issues. The three-layer results of T1 and T4 are considered to be imprecise, as the measurement was carried out at the flow boundary of 30 L/min, which was the defined upper boundary for breathability (triple-layer, red cross-hatched bars). The double- and triple-layer configurations for T11 were found to be above the defined flow rate (bars are not included).

This result can be explained by the following. In a double-layer configuration, the weave pattern alignment roughly halves the effective area for breathing and roughly doubles the area where the particles can get stuck. The efficiency of the breathability can also depend on the alignment of the weave patterns, i.e., the gaps and threads of the samples. However, the deviances were not significantly different for single- and multilayer cases. By fitting the layers with misalignment in the X or Y direction, the intersecting mesh structure of the fabrics produced a sufficient overlap—thus improving the filtering efficiency significantly. The third layer could not significantly increase this, but further improvements can still be achieved. The analysis of the overall filtration efficiency value revealed that the fabric also trapped smaller particles more efficiently. Investigating the complex structure of the triple-layer configurations is a task that was beyond the time constraints of the current work, but 3D simulations can be carried out for further investigation, similar to the ones found in [9].

**Figure 4:**
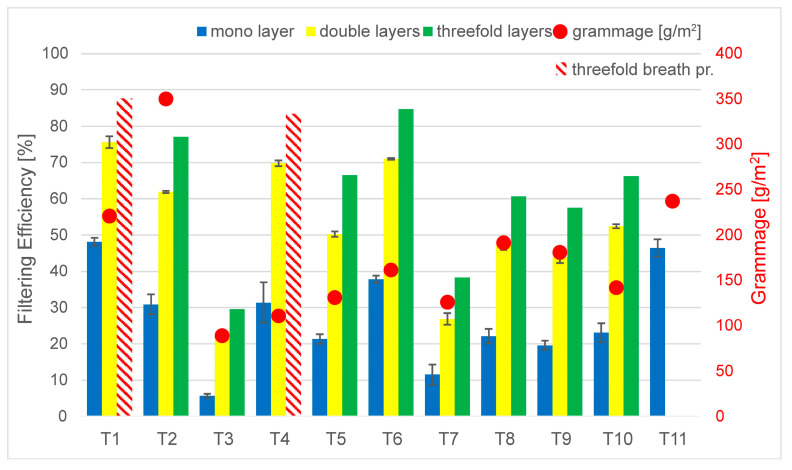
Filtering efficiency versus grammage.

**Figure 5:**
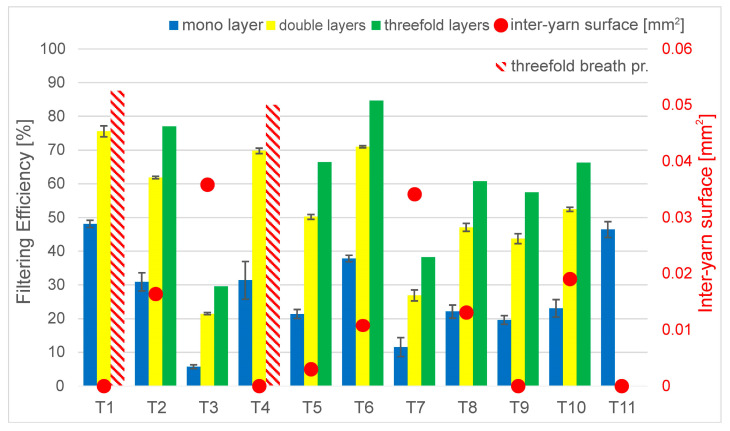
Filtration efficiency versus inter-yarn surfaces.

The comparison between the grammage and filtering efficiency (Figure 4) shows that larger fabric weight generally led to better filtration, especially when more layers were considered. The lowest filtration occurred with T3 and T7, which had lower grammage. However, the grammage of T4 was in a similar range, but its filtration efficiency was much better. While the correlation is not straightforward, it is suggested to perform fabric weight measurements when considering filtration efficiency during the construction of new mask types and to continue the investigations with the aspects presented in the following figures in the chapter. Comparing the inter-yarn pore surface results with the filtration efficiencies (Figure 5) in line with results found in Section 3.1, the findings also present valuable information. It can be said that two of the loosest weaves had very low filtering efficiency, as there was a clear connection between the pore surfaces and filtration. Additionally, it is important to note that the inter-yarn pore surfaces were in the range of 1–10 µm, and the obtained results are slightly improved compared to those in [9].

In addition, it is also worth mentioning that the average standard deviation was about two percent, so the measurement is reproducible, and the materials were homogeneous. Additionally, practically wherever the sample was prepared from the available sheets, the results were consistent with the other measurements. The results are also in line (albeit extended) with those found in [6], where the filtering efficiency remained below 60% for the majority of the community-used, commercial masks tested (which were specifically designed for protection).

### 3.3. Statistical Analysis

A single-factor variance analysis of the results proved that there were statistically significant differences between the samples at a significance level of 0.05. It was also found with Tukey’s test that the following groups could be formed. The efficiency of single-layer filtration is the same for the following groups named by the following convention: GX (G—group, X—group number): G1 (T1, T11), G2 (T2, T4, T6), G3 (T5, T8, T9, T10), and G4 (T7, T3). For two layers, the following groups can be formed: G5 (T1, T11), G6 (T4, T6), and G7 (T5, T10). The remaining samples differed from each other and were not grouped. In conclusion, cotton twill and canvas were very similar, and satin-weave viscose and natural silk, and plain-weave cotton and plain-weave cotton sheet can be considered similar in terms of filtration.

It is important to note that according to the Pearson coefficients, there was no correlation between the filtration efficiency (FE) and the grammage or mesh (XY). However, there was a clear correlation between the pore surface size and filtration (highlighted as “Inter-yarn”). Table 3 shows the correlation coefficients.

## 4. Discussion

The relation between the inter-yarn pore surface size and the filtration efficiency had a high negative correlation value with a range of similar significance, indicating that if the surface increases, the filtration efficiency is reduced. This strong correlation confirms that textiles with large inter-yarn surfaces are not appropriate for personal protection. Additionally, inter-yarn gaps had a weak correlation with the grammage and mesh parameters.

**Figure 6:**
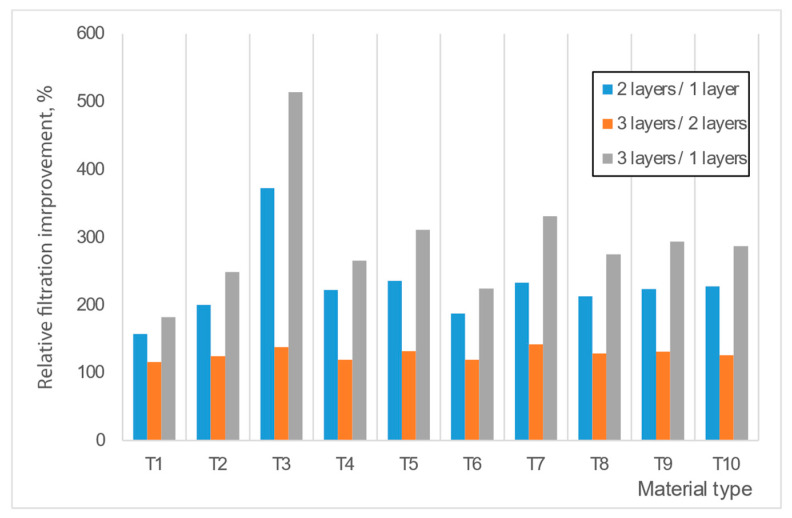
Improvement ratio over layer stacking.

Figure 6 and Table 4 reveal the relative filtration improvement (increased filtration of X layers/original filtration of X layers, %, where X notes the number of layyers) over layer stacking. The filtration efficiency improved considerably when applying a second layer onto the first layer. The improvement was around a 1.5-4-fold increase. The improvement from two to three layers was less significant. According to the statistical analysis and the obtained Pearson’s coefficients (Table 4), the double-layer application is highly recommended for most materials, while triple-layer application, with its breathability problems, is not necessarily recommended.

The measurement method presented here was found to be suitable for the qualitative and quantitative filtering efficiency testing of different low-cost fabrics. Double-layer configuration improved the filtering efficiency significantly, whereas triple-layer configuration led to practical problems with breathability. Natural silk, satin-weaved viscose, and handmade linen are recommended as mask materials for double (or, with overall ergonomic limitations, triple) layering, while the other options did not reach the level of a medical mask.

**Figure 7:**
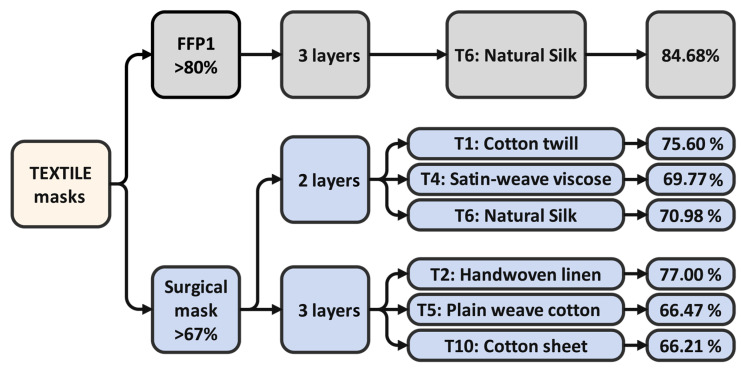
Recommendation for applications with given filtration efficiencies.

Figure 7 summarizes the recommendation for the given textile material set, highlighting a possible outcome for usable fabrics. It is important to note that that only one material was able to achieve the level of FFP1—this was the T6, the natural silk with three layers. Six other textiles were able to reach the surgical mask level with the double- or triple-layer configuration. These were satin-weave viscose, cotton twill, and natural silk with two layers, and handwoven linen, plain-weave cotton (printed blue), and plain-weave cotton sheet with three layers.

A further step could be to explore the possibilities of combining different materials. A further future goal could be to investigate the effects of different cleaning procedures or washing on the degradation of the filtration value [16]. Both points are our future focus. The current findings can be used in smart mask applications [24], where active electronics work together with filtering materials and sensors to investigate outbreak spread. Modeling of particulate spread and dispersion is also a path for the expansion of our findings [25]. Future works can also investigate the aspect of the health index, with a focus on national relations.

## 5. Conclusions

In this study, we investigated a problem with global societal concern; we applied a novel measurement method to test low-cost, affordable, and sustainable cloth-based textile materials as mask materials, qualitatively and quantitatively describing the cloth-based solutions often used during the COVID-19 pandemic. During the measurements, we investigated 11 types of cloth textiles prepared for the sustainable production and consumption of protective masks.

Summing up the results, natural silk with three layers was able to produce a level of filtration efficiency of 84.68%, which is similar to FFP1 masks, with reasonable breathability. For the level of a medical mask, with two layers, natural silk achieved filtration of 70.98%, exceeding the required 67%; cotton twill with two layers achieved 75.6%, and satin-weave viscose achieved 69.77%. When three layers were applied, the handwoven linen canvas reached an adequate level of 77%; plain-weave cotton (printed blue) (66.47%) and plain-weave cotton sheet (66.21%) are also options to be considered in cases where lower filtration is acceptable. It was statistically shown that applying a second layer was more significant from the overall filtering aspect than increasing the layer count to three.

It can be concluded that the relationship between the grammage and mesh count was not significant; the statistical results show no significant correlation them. What is emphasized by the statistical analysis is that materials with fewer free surfaces (gaps) were generally better filtering materials.

Handmade masks with carefully selected materials and fabric types (different weave or knitting patterns) in multilayer configurations can at least reach the filtering efficiency level of medical masks—as our findings suggest. However, single-layer masks made at home from commercially available cloths are generally not suitable for filtration even to the lower standard. Their use should be avoided if a better solution is available, or it is suggested to combine the layers for improved application. It is suggested that further treatment of the materials (e.g., washing, disinfection [26]) or increasing the breathing volume flow further reduces their performance, so application in moderate to high activity (sports, demanding manual work) is not suggested.

Based on the literature and the WHO recommendations, applying a poor filtering material is still a better alternative than not wearing a mask at all during critical moments of the epidemic—but the limitations must be taken into consideration.

## Figures and Tables

**Table 1:** Textile materials used in the experiments.

Textile No.	Textile Material Type
**T1**	Cotton twill
**T2**	Handwoven linen
**T3**	Linen canvas
**T4**	Satin-weave viscose
**T5**	Plain-weave cotton (printed blue)
**T6**	Natural silk
**T7**	Mercerized cotton canvas (floral printed)
**T8**	Knitted cotton fabric (white)
**T9**	Knitted cotton jersey (red)
**T10**	Plain-weave cotton sheet
**T11**	Cotton canvas (red)

**Table 2:** Optical inspection of different textile samples.

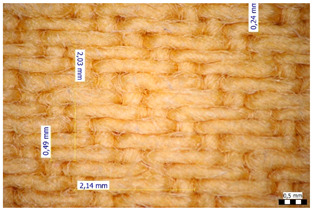	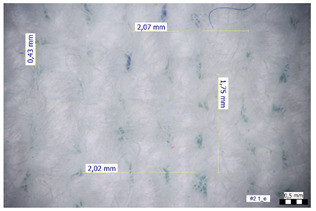
T1—Cotton twill.	T2—Handwoven linen.
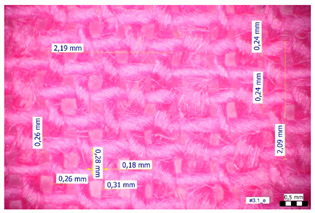	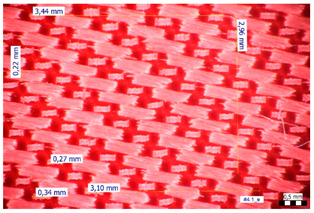
T3—Linen canvas.	T4—Satin-weave viscose.
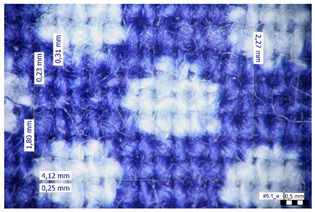	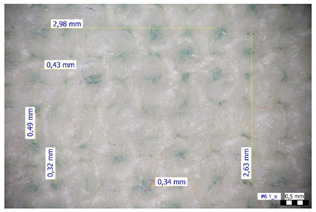
T5—Plain-weave cotton (printed blue).	T6—Natural silk.
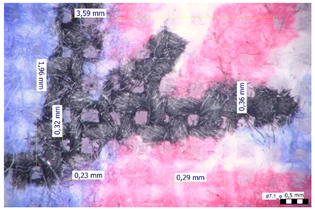	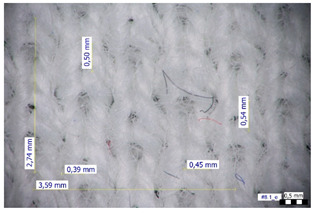
T7—Mercerized cotton canvas (floral print).	T8—Knitted cotton fabric (white).
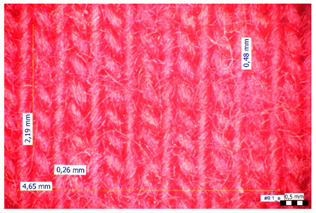	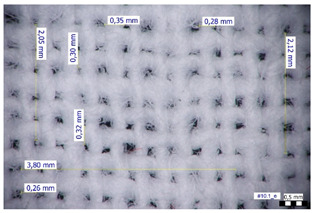
T9— Knitted cotton jersey (red).	T10— Plain-weave cotton sheet.
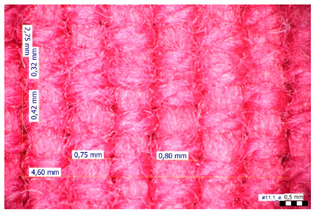	
T11—Cotton canvas (red).	

**Table 3:** Correlation between different material parameters.

Param.	FE 1 Layer	FE 2 Layers	FE 3 Layers	Gramm.	Mesh (X)	Mesh (Y)	Inter-Yarn
Gramm.	0.545415	0.449036	0.466829	1			
Mesh (X)	−0.01885	0.255545	0.244196	−0.39069	1		
Mesh (Y)	0.134714	0.310559	0.301102	−0.4443	0.373181	1	
Inter-yarn	−0.70685	−0.74309	−0.766599	−0.23928	−0.37844	−0.43188	1

**Table 4:** Pearson coefficients of layer stacking.

	2 Layers/1 Layer	3 Layers/2 Layers	3 Layers/1 Layer
**2 layers/1 layer**	1		
**3 layers/2 layers**	0.671	1	
**3 layers/1 layer**	0.989	0.765	1

## Data Availability

Data is available upon request.
